# Nigerian and Ghanaian Young People’s Experiences of Care for Common Mental Disorders in Inner London: Protocol for a Multimethod Investigation

**DOI:** 10.2196/42575

**Published:** 2022-12-09

**Authors:** Anthony Isiwele, Carol Rivas, Gillian Stokes

**Affiliations:** 1 Social Research Institute Institute of Education, University College London's Faculty of Education and Society University College London London United Kingdom

**Keywords:** Nigerian, Ghanaian, lived experience, common mental disorder, mental health, London, mental healthcare, mental disorder, ethnic, minority, racial, preference, perspective, patient need, qualitative, experience, content analysis, mental illness, phenomenological, phenomenology, policy analysis, United Kingdom, Great Britain, youth, pediatric, adolescent, adolescence, young person, young people

## Abstract

**Background:**

The Care Quality Commission published a review in 2018 in England titled “Are We Listening,” which revealed that child and adolescent mental health services are not responsive to the specific needs of young Black people and other ethnic minorities even in areas with ethnically diverse populations. It found that commissioners and service planners failed to engage with these young people and their families to understand their needs and expectations.

**Objective:**

The purpose of this study is to engage Nigerian and Ghanaian young people (NAGYP) with experiences of care for common mental disorders (CMDs) in London, to increase understanding of their needs, and to give voice to their views and preferences. Their parents’, caregivers’, and practitioners’ views will also be sought for service improvement.

**Methods:**

Three combined contemporary complementary methodologies—thematic analysis, interpretative phenomenological analysis (IPA), and intersectionality-based policy analysis (IBPA)—will be used across 3 comprehensive phases. First, a scoping review where relevant themes will be critically analyzed will inform further phases of this study. Detailed mapping of community and mental health care services in 13 inner London boroughs to investigate what professionals actually do rather than what they say they do. Second, IBPA will be used to scrutinize improving access to psychological therapies and other legislations and policies relevant to NAGYP to undertake an intersectional multileveled analysis of power, models, and constraints. Third, IPA will “give voice” and “make sense” of NAGYP lived experiences of CMDs via a representative sample of NAGYP participants’ (n=30) aged 16-25 years, parents or caregivers’ (n=20), and practitioners’ (n=20) perspectives will be captured.

**Results:**

The study has been approved by the UCL Institute of Education Research Ethics Committee (Z6364106/2022/02/28; health research) and University College London (Z6364106/2022/10/24; social research). Recruitment has begun in 13 inner boroughs of London. Data collection through observation, semistructured interviews, and focus groups are expected to be finalized by early 2024, and the study will be published by early 2025.

**Conclusions:**

Combining multiple qualitative methodologies and methods will enable rigorous investigation into NAGYP’s lived experiences of care received for CMDs in London. Findings from this study should enable a reduction in the negative connotations and harmful superstitions associated with mental health–related issues in this group, inform evidence-based interventions, and facilitate preventive or early access to interventions. There may also be an indirect impact on problems resulting from mental illness such as school dropout, antisocial behaviors, knife crimes, juvenile detention centers, and even death.

**International Registered Report Identifier (IRRID):**

PRR1-10.2196/42575

## Introduction

### Background

Since the 1970s, there have been concerns that the mental health system in the United Kingdom does not lend itself to the specific needs of Black people [1-3]. This may have led to the establishment of the Nafsiyat Intercultural Therapy Centre for ethnic minorities in London in 1983 [4,5]. Yet in 2018, the Care Quality Commission, an independent regulator, published its findings from a review of England’s child and adolescent mental health services (CAMHSs) entitled “Are We Listening?” [6]. This revealed that, even in areas with ethnically diverse populations, CAMHSs are still not responsive to the specific needs of Black young people and other minorities. They found, “commissioners and service planners had failed to engage with...young people, families, and caregivers to understand their needs and expectations” [6]. Direct engagement with ethnic subgroups is a knowledge gap that this study aims to fill.

Thus, this study aims to engage a section of 2 underserved communities, Nigerian and Ghanaian young people (NAGYP), to increase understanding of their care needs for common mental disorders (CMDs) in inner London. The study will also engage with their parents or caregivers and practitioners to capture their views on CMDs and mental health care (MHC) models. It starts from the position that MHC needs to be reflective of cultural humility toward NAGYP as conceptualized in multicultural competencies [7,8].

This is the first study in the United Kingdom to explore NAGYP mental health experiences as part of the push against a one-size-fits-all approach to MHC [9-11]. In the domain of ethnic minorities, Lavis [9], Butt et al [10], and the London Assembly [11] highlight that paying specific attention to the needs of different subgroups and individualization is paramount. Vostanis et al [12] in their work on Indian adolescents in England argued, “rather than a blanket approach being applied to policy and service planning to meet the needs of diverse communities of young people, more specific evidence needs to be gained.” Given the size of the NAGYP population in the United Kingdom, this NAGYP study will add to the body of evidence.

The 2011 Census analysis for ethnicity (as we wait for the 2021 Census, due to be published in early 2023 [13]) estimated that 312,000 Nigerian-born and 130,000 Ghanaian-born people live in the United Kingdom [14]. It also showed that around one-fifth of the foreign-born population of England and Wales was born in Africa (1.3 million, 17%). Those from Ghana and Nigeria had the highest proportion of Black or Black British (both 89%, 285,000) people [15]. London is the setting of this study, with a 2020 population of 9 million [16], and of the non-UK born, London has a Nigerian and Ghanaian population of 135,000 and 63,000, respectively [17]. These population sizes are much larger than those of the Jewish community (n=7770) in the City of Salford, Greater Manchester [18], or the Chinese community (n=14,000) in Northeast England [19], whose young people have already been the subject of research.

### Prevalence of CMDs Varied by Ethnicity

The 2014 age-standardized data showed that on average, Black people are more likely to report a CMD: Black and Black British 23%, mixed and other 20%, Asian and Asian British 18%, White British 17%, and White other 14% [20,21]. For children and young people, the latest series (2017, 2020, and 2021) of mental health surveys sponsored by the Department of Health and Social Care did not show CMD prevalence by ethnicity. For all ethnic groups, CMD rates were higher in girls (10%) than boys (6.2%) in 5- to 19-year-olds [22,23]. The data for ethnicity was on general mental disorders; among 6- to 23-year-olds, White British, mixed or other, Asian or Asian British, and Black or Black British people were estimated at 18.9%, 22.5%, 8.4%, and 8.3%, respectively [23].

There are a few issues to consider regarding the lack of data and its inconsistencies. First, the data for CMD prevalence by ethnicity is grossly limited. There is evidence of variation in CMD prevalence and symptom presentation among ethnic subgroups [24,25]. The London Assembly [11] was unequivocal that nuanced data on ethnic subgroups “simply does not exist.” When the nature and scale of the demand for mental health services are not known, it inhibits policy makers’ and service planners’ responses. The Assembly emphasized the frustration of funding and commissioning services with little or no knowledge of the demand for those services.

Second, the data suggest links between poor mental health, youth, and gang violence [26-28]. This has led to young Black people being wrongly associated with a criminal proclivity, rather than this being acknowledged as the result of structural inequalities. A practitioner participant in Fitzpatrick et al’s [29] study said, “When I became a consultant [...] I saw Black people..., not being given the more respectable diagnoses but the more derogatory ones, those that carry punishment instead of therapy.”

### Definition of Common Mental Disorder

The British Psychological Society and The Royal College of Psychiatrists recognize “depression” (including subthreshold disorders) and “anxiety” (including generalized anxiety disorder, panic disorder, phobias, social anxiety disorder, obsessive-compulsive disorder, and posttraumatic stress disorder) as CMDs [30]. In some works of literature, depression and anxiety disorders are recognized and are often grouped together as “emotional disorders” as a more restrictive definition of CMDs [9,11,31,32]. CMDs has been chosen as the acronym or term in this protocol to align with the National Collaborating Centre for Mental Health and National Institute for Health and Clinical Excellence definitions.

### Study Aims and Objectives

The primary aim is to investigate the NAGYP experiences of MHC for CMDs in inner London in order to give voice to their views and preferences for service improvement. The study has 5 key objectives:

1. To identify the care and treatment options available for NAGYP in London living with CMDs
2. To evaluate how culturally appropriate and potentially adaptable the Positive Practice Guide of Improving Access to Psychological Therapy (PPG-IAPT) is for NAGYP service users, which is the first line of treatment for CMDs
3. To investigate the lived experience of NAGYP of care for CMDs in inner London and the views of their parent or caregiver on the construct of CMDs
4. To ascertain how practitioners use models in their repertoire to care for NAGYP
5. To understand how NAGYP’s views, preferences, and expectations could inform care and practice design

## Methods

### Combining Thematic Analysis, Interpretative Phenomenological Analysis, and Intersectionality-Based Policy Analysis

This study has chosen 3 contemporary complementary methodologies to achieve the research objectives at different phases. This choice stems from the consideration of CMDs as a phenomenon and their impact on the NAGYP social world. These philosophical underpinnings reflect the personal and professional background of the lead researcher as a Black man and a social worker, and the sensitive nature of the phenomenon, both in terms of cultural stigmatization [11,25] and institutional mistrust [33] that characterizes NAGYP’s reality.

**Figure 1:**
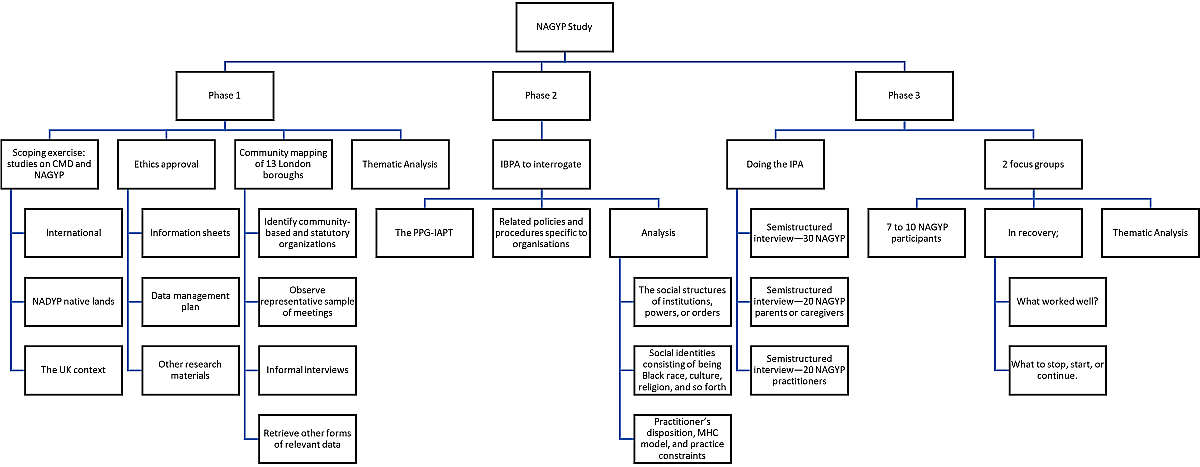
Research design for NAGYP London. CMD: common mental disorder; IBPA: intersectionality-based policy analysis; IPA: interpretative phenomenological analysis; MHC: mental health care; NAGYP: Nigerian and Ghanaian young people; PPG-IAPT: Positive Practice Guide of Improving Access to Psychological Therapy; TA: thematic analysis.

While thematic analysis (TA) will focus on NAGYP’s lived experiences in terms of what a CMD as a phenomenon “looks like,” interpretative phenomenological analysis (IPA) [34] will focus on what it “feels like” [35]. In context, TA will culminate in “deeper-level analysis relating to power and communities” [35], and on the other hand, IPA will “give voice” to and “make sense” [36] of NAGYP’s own accounts. In addition, intersectionality-based policy analysis IBPA [37] will focus on intersectional issues, particularly how NAGYP social identities related to race, culture, religion, status, and so forth, and dispositions intersect with shared social structures and context [38,39]. IBPA will be used to scrutinize the PPG-IAPT for NAGYP constituents in order to expose the assumptions that characterize policy formulation in the absence of robust direct engagement with those for whom the policies are intended [40,41]. The study will be undertaken in 3 comprehensive phases, see Figure 1.

### Phase 1: Scoping Exercise and Community Mapping

#### Scoping Exercise

In the United Kingdom and England in particular, there is a body of work on CMDs in relation to ethnic minority children and young people [29] (eg, [42-46]). However, little is known about Black or African people specifically [25] (eg, [47,48]), and very little or nothing is known about the individual or combination of this study’s subgroups (Nigerian and Ghanaian), geographical location (London), and their relationship with the phenomenon (CMDs). Thus, this phase will review literature that examines elements of NAGYP and Black African people with experiences of CMDs. Boote and Beile [49] suggest that for topics about which little or nothing has been written, the reviewer may need to “broaden the search” to explore related topics. Therefore, the principles of a scoping review will be used to “determine the scope or coverage” [50]. Cooper [51] argues that “coverage” is the most distinct element of a literature review. Thus, the coverage will include NADYP native lands and UK studies, though with a particular focus on London as the primary geo-socio-political context of this study. Relevant themes will be critically analyzed and will inform further phases of this study.

#### Studies on the CMD Construct Related to the NAGYP Domain Globally

The rationale and understanding of the impact of CMDs on Black African young people at a global level will be important to place our UK findings in context. Attention will be given to the location and social context from which samples were drawn.

#### Studies in NAGYP Native Countries

Relevant studies undertaken in Nigeria and Ghana will be synthesized. Findings from NAGYP homelands will increase understanding of the perceptions of NAGYP, their general disposition, and what they make of CMDs. This is crucial because when people migrate, they do so with the health perceptions and cultural and religious beliefs developed within their country of origin.

#### Studies in the United Kingdom

In the United Kingdom, since health is a devolved matter across the constituent countries, relevant literature will have a national spread across England, Scotland, Wales, and Northern Ireland. However, the literature from England, specifically London, will, where possible, have primacy in informing further stages. London is the primary social, political, and environmental context of this study, where the researcher will engage in multimethod research activities with NAGYP, parents or caregivers, and practitioners.

#### Databases

The selected databases and libraries include International Bibliography of the Social Sciences, Applied Social Sciences Index and Abstract, Web of Science, SCOPUS, UCL Explore, Google Scholar, and Academic Search Complete (via London Senate House Library). These databases host a rich variety of social science peer-reviewed literature with international coverage. In addition, studies will be added through snowballing from the included studies’ reference lists [52].

#### Ethics Approval

Ethical approval has been granted by the UCL Institute of Education Research Ethics Committee (Z6364106/2022/02/28; health research) and University College London (UCL) (Z6364106/2022/10/24; social research). We are waiting for approval from the National Health Service (NHS) Health Research Authority Ethics Committee.

#### Community Mapping

Rigorous community mapping will be undertaken in these inner London boroughs: Camden, Greenwich, Hackney, Hammersmith and Fulham, Islington, Kensington and Chelsea, Lambeth, Lewisham, Southwark, Tower Hamlets, Wandsworth, Bexley, and Westminster. These have been chosen from among London’s 32 boroughs because they are home to a sizable NAGYP population [53]. There are 5 objectives in community mapping within London boroughs. They are (1) to identify community-based organizations delivering specific MHC services to NAGYP or Black African people or minority groups within them, (2) to ensure voluntary and statutory organizations with departments providing such services are included within them, (3) to attend a representative sample of meetings where relevant care is being discussed or delivered, (4) to conduct an informal interview with a representative sample of practitioners, and (5) doing TA with a framework approach.

The objective is to investigate the ways and extents to which MHC and elements of models are actually adapted by practitioners to meet the specific mental health needs of NAGYP. Contacts will be made directly with these departments or teams. Permission will be sought to attend and observe selected meetings, at least one from each borough, where care plans are discussed, as well as workshops or sessions where care is delivered in action. The ones deemed appropriate will be attended to with due regard to full ethical and governance requirements. If permission is not granted, data from the relevant service websites would be analyzed instead. Some practitioners (n=20-25) will also be invited for an informal interview at this stage.

#### Informal Interviews

Various spontaneous, unscheduled interviews will be undertaken with practitioners after meetings or observed sessions, either to clarify or better understand certain approaches or practices. These informal interviews are meant to complement what the researcher observes as part of ethnographic interviews [54]. This has the potential to validate what is discussed in meetings, adding to the authenticity of the data. The interview content will be written in a field notebook as soon as possible, as it may not have been recorded due to the spontaneous nature of the interview [54]. Informants may provide different amounts of information depending on what the researcher needs to know. To manage bias, the information collected will be incorporated as field note data rather than interview data.

#### Doing TA With a Framework Approach

**Figure 2:**
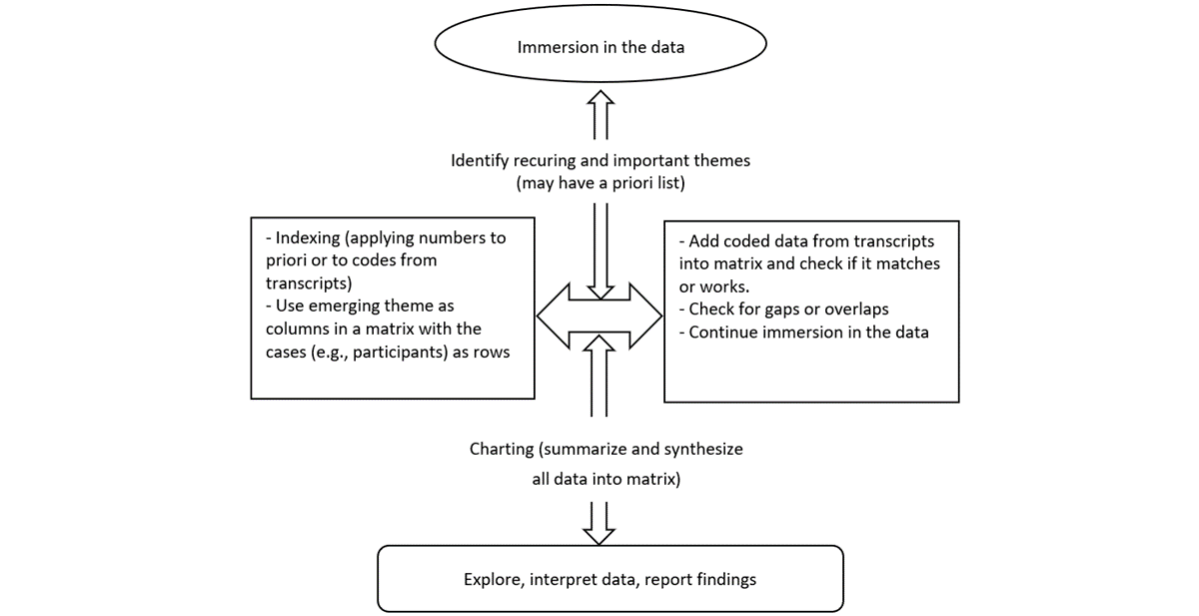
Stages of the framework approach [35].

The qualitative data generated in this phase will be analyzed using the framework approach. Framework matrices (called charts) enable the analysis of data from a wide variety of sources, such as websites, PDFs, audio or video recordings, blog posts, field notes, memos, and transcripts generated from informal interviews. The approach allows all data to be collected before analysis begins. The data will be imported into NVivo (QSR International), where it will be summarized in charts according to predetermined themes [55]. The reporting for TA is normally written in a descriptive list, as “careers” or journeys through time or place, or as a typology. This study will be written in a descriptive list format to ensure that findings are communicated in a concrete (rather than more conceptual) manner that is understandable to wider audiences [35]. The process is illustrated in Figure 2.

### Phase 2: Interrogating the PPG-IAPT for Black People and Ethnic Minorities

The IBPA will be used to scrutinize the PPG-IAPT as the main MHC national policy, as well as other organizational internal policies and procedures for NAGYP and its constituents with CMDs. IBPA is an “equity-promoting public policy analysis” framework [37] that will be used to perform a multileveled intersectionality analysis. The analysis will emphasize the simultaneous interplay and triangulation between:

1. The social structures of institutions, powers, or orders on one strand
2. Social identities consisting of being Black race, culture, religion, and so forth, as the second strand
3. Practitioner’s disposition, MHC model, and practice constraints as the third strand

**Figure 3:**
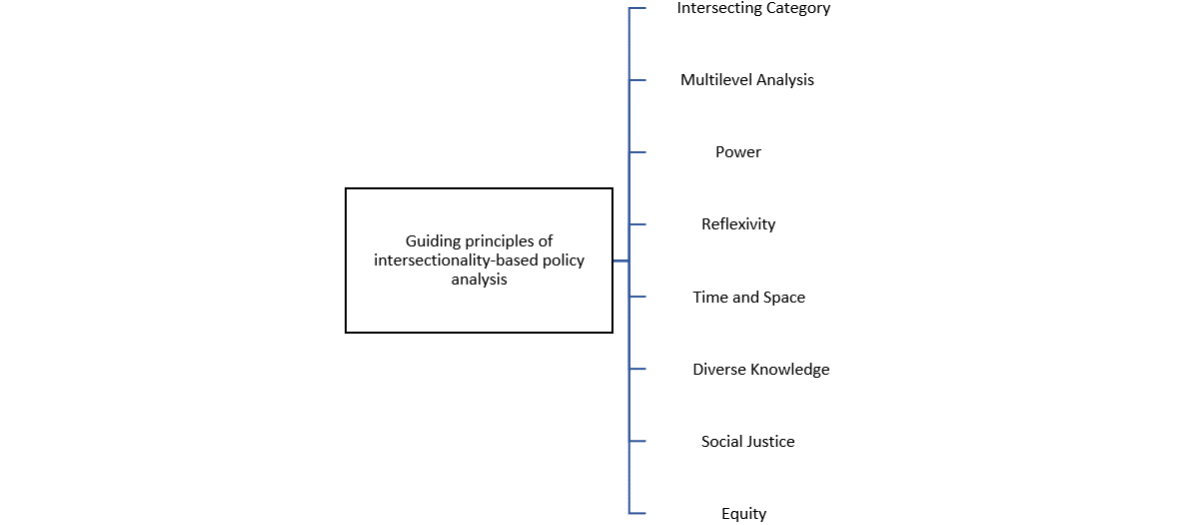
Guiding principles of intersectionality-based policy analysis [37].

The aim is to ascertain the PPG-IAPT and the related policy of its cultural adaptiveness and appropriateness to the specific needs of NAGYP service users. The 2 core components of IBPA will be used. They are (1) a set of guiding principles (see Figure 3) and (2) a list of 12 overarching questions (see Textbox 1) [37] with a set of subquestions [56].

The design is for the principles to be used with the questions (including the subquestions) simultaneously. Each question will be asked and answered in a manner that would depict explicit intersectionality-informed analysis. The aim is to draw attention to those assumptions that characterize policy formulation without robust direct engagement with those whom the policies are intended for [40,41].

The 12 questions are divided into 2 categories; the first 5 are termed “descriptive.” This will expose the critical background information about the problem in the PPG-IAPT and its related policies for ethnic minorities. This phase will pay particular attention to how the problems the policy is meant to ameliorate are identified, deconstructed, and then addressed. For example, only 1 Nigerian and no Ghanaian people were involved in the focus group in the formulation of the PPG-IAPT. This will bring to light the assumptions as well as the inequities or privileges, if any, that inundate the policy position. The remaining 7 questions are termed “transformative.” These are intended to help identify alternative policy responses or proffer suitable solutions that could provoke social and structural change. Phase 1 will play a fundamental role in this. The overarching goal of IBPA is to reduce inequities, if not completely eradicate them, and ultimately to promote equity and social justice [37].

Hankivsky et al [37] argued that “simplicity and flexibility are key features of the Framework.” All 12 questions may not be relevant to this study. For this study, some of the questions may be prioritized and given more consideration than others due to the context and approaches to implementation adopted by individuals or teams. What is critical is that the questions are rooted in key intersectionality principles subsumed in structure and politics [39]. The combined effect of these categories of questions on the PPG-IAPT for minority groups as a national policy position and its related policies and procedures unique to individual organizations would transform how its associated problems and processes are understood.

**Descriptive and transformative questions of intersectionality-based policy analysis. Adapted from Hankivsky et al [37].**

**Descriptive questions**

1. What knowledge, values, and experiences do you bring to this area of policy analysis?
2. What is the policy “problem” under consideration?
3. How have representations of the “problem” come about?
4. How are groups differently affected by this representation of the “problem”?
5. What are the current policy responses to the “problem”?

**Transformative questions**

1. What inequities actually exist in relation to the “problem”?
2. Where and how can interventions be made to improve the “problem”?
3. What are the feasible short-, medium-, and long-term solutions?
4. How will proposed policy responses reduce inequities?
5. How will implementation and uptake be assured?
6. How will you know if inequities have been reduced?
7. How has the process of engaging in an intersectionality-based policy analysis transformed:
  Your thoughts on relations and structures of power and inequity?
  The way you and others engage in policy development, implementation, and evaluation?
  Broader conceptualizations, relations, and effects of power asymmetry in the everyday world?

### Phase 3: Engaging With NAGYP, Parents, and Practitioners

This is the core of the study and provides, through semistructured interviews, an in-depth understanding of the lived experiences of NAGYP on the MHC received for CMDs. Their parents’ and practitioners’ views are a key part of this. The focus of this is to use IPA to “give voice” and “make sense” [36] of NAGYP’s experiences in their own words, terms, and accounts without affiliation to any existing theory or concept [34].

#### Semistructured Interviews

##### Semistructured Interview: NAGYP

Around 25 to 30 NAGYP participants in the age range of 16-25 years will be identified and recruited from the phase 1 exercise. In addition, participants will be recruited through local gatekeepers such as notable voices, faith and community leaders, local associations, and voluntary service providers within the community. The interview will explore themes related to the following topics:

- Meaning and perception of CMDs
- Experience during therapy
- Views, preferences, and expectations
- Anything particularly helpful in MHC
- What to stop, start, or continue

##### Semistructured Interview: NAGYP Parents or Caregivers

This is aimed at capturing about 15-20 NAGYP parents’ views and perceptions of the CMD and MHC constructs. Topics will aim to enable the following:

- Reducing the negative connotations and harmful superstitions of CMDs that characterize NAGYP communities
- A more liberal understanding of CMDs
- Improving access to early intervention or professional help

Parents and caregivers will be interviewed because the meaning they attach to CMD discourses, negative or positive, is often passed on to their young people due to the strong family ties that exist among Black parents, children, and young adults resulting from their cultural dispositions of the family unit [57,58].

##### Semistructured Interview: NAGYP Practitioners

The interviews will be designed to elicit the practitioner’s practical knowledge of MHC models. They are aimed at practitioners in the selected London areas who have delivered MHC to NAGYP. Up to 15-20 participants will be identified from phase 1. One practitioner will be included in an embedded pilot. The knowledge generated from this research activity would be used to categorize the different practitioners’ understandings of MHC models relating to the services they provide for NAGYP as well as the options available within and across disciplines. The dimensions of the categorization will be substantiated in the domain of MHC models in existing literature, as currently understood. However, their preference for modification or for new MHC models for NAGYP will be benchmarked against the medical and social models of disability [59] and Eurocentric and Afrocentric MHC. The interview topic guide includes:

- The most prevalent CMD diagnosis for NAGYP service users
- Own experience as a therapist supporting NAGYP
- Professional training received in response to meeting the needs of NAGYP, or Black young people in general
- Perception of the most suitable model of intervention
- Effective ways of integrating the model with the PPG-IAPT
- Challenges and key determinants of success in providing MHC to NAGYP
- Potential examples of positive practice
- The future of MHC for NAGYP

#### Doing the IPA

This phase will culminate in the IPA protocol. The most recent changes to the terminology of IPA will be adopted in the analysis. For example, the usual emergent themes and superordinate themes will be called experiential statements and personal experiential themes, respectively [60,61]. The following steps in Textbox 2 will be adhered to.

**Steps in doing interpretative phenomenological analysis. Adapted from Smith et al [61].**

Step 1: Starting with the first case: reading and rereading, be immersed in the transcript. This is to make sure the respondent becomes the focus of the analysis. Similar to thematic analysis.

Step 2: Exploratory noting: disentangling semantic content, language, and conceptual comments with an open mind, noting everything of interest, and developing an avowedly interpretative statement relating to context. This will be reviewed with my supervisor.

Step 3: Constructing experiential statements: the process of consolidating and crystallizing the exploratory notes. This process represents 1 manifestation of the hermeneutic circle, that is, “the me” and the lived experiences of the participant in collaborative (cocreating) efforts. Tied within local instances in the transcript.

Step 4: Searching for connections across experiential statements: clusters of statements can be organized through different possibilities, using flexibility.

Step 5: Naming the personal experiential themes (PETs), consolidating, and organizing them in a table. Not tied within local instances but within the transcript as a whole.

Step 6: Continuing the individual analysis of other cases: steps 1-5 will be repeated for other cases in their own terms and individuality, in keeping with IPA’s idiographic commitment.

Step 7: Working with PETs to develop group experiential themes (GETs) across cases: drawing links between each PET to create GET.

#### Focus Group

To pay greater attention to the views and preferences of NAGYP toward CMDs and MHC to inform care and practice design, focus groups will be used to engage with NAGYP. Participants will be recruited through the established contacts from phase 1. In the focus group discussion, 7 to 10 NAGYP participants in the age range of 16 to 25 years will be involved. The topic guide will be informed by the data collected so far; 2 sessions are anticipated. The activities will be documented and analyzed using standards for handling data from multiple voices [62,63]. The focus groups will explore the experiences of MHC; the experiences that play a substantial role in recovery; what worked well; and what to stop, start, or continue.

## Results

The study has been approved by the UCL Institute of Education Research Ethics Committee (Z6364106/2022/02/28; health research) and UCL (Z6364106/2022/10/24; social research). Recruitment has begun in the 13 inner boroughs of London. Data collection through semistructured interviews and focus groups is expected to be finalized by early 2024, and the study will be published by early 2025.

## Discussion

The study aims to investigate NAGYP’s lived experiences of care for CMDs in inner London. The study anticipates identifying the care and treatment options available; the cultural appropriateness of the PPG-IAPT for NAGYP service users, which is the first line of treatment; and parents’ views and practitioners’ dispositions on models of care. We hope the outcomes of this study will contribute to providing a response to the London Assembly’s recognition of mental disorders as a peculiar problem being faced by young Londoners, particularly from minority ethnic subgroups. The Assembly acknowledged how this could negatively impact their well-being and economic capacities [12]; some impacts may lead to antisocial behaviors and fatalities [26,27]. We also expect the findings to be consistent with the recommendations in the joint Green Paper published by the Department of Health and Social Care and Department for Education [64]. The Green Paper captured the views and expectations of 65 respondents from Black or minority ethnic backgrounds and LGBT+ communities, or those who have a disability; all were younger than 25 years. Their expectations were unequivocal, including creating a welcoming environment, training the CAMHS workforce to gain cultural competence skills, providing bespoke MHC, improving service awareness, and providing out-of-term time support [65]. These expectations highlighted in the Green Paper have not yet been met; a report published in February 2022 by the NHS Race and Health Observatory body found evidence of ethnic inequalities in every area reviewed [65].

The NHS Race and Health Observatory is an independent expert body given the responsibility of examining health inequalities experienced by minority ethnic groups in England, of which NAGYP is a major constituent. Their main findings in a review revealed that Black people’s fear and distrust of mental health services form “clear barriers to seeking help” [66]. Thus, a key strength of this study is in the bottom-up and transparent methodological choices. For example, while TA interrogates NAGYP’s lived experiences of what CMD as a phenomenon looks like and how it feels like, IPA allows idiographic accounts in participants’ own words and terms to allow the very essence of the phenomenon to reveal itself in its primordial form [36]. Then, NAGYP’s social identity and context are explored within the precepts of IBPA.

The main potential limitation, while also a richness, is that qualitative methods are focused mainly on participants’ experiences [67,68] and seek to gain access to participants’ social worlds. As a result, Smith et al [66] acknowledge that IPA researchers might strongly influence the interpretation of the respondent’s world. According to Larkin et al [36], the wide range of interpretative frameworks available for IPA constitutes a practical problem. Cromby and Nightingale [69] noted that the part of the participant’s world that the researcher would want to make real or relative may be typically dependent on choices shaped by the researcher’s “moral, political or pragmatical precepts” instead of epistemological choice. Larkin et al [36] put it succinctly that “we can never fully escape the ‘preconceptions’ that our world brings with it,” thus, transparency is key. Therefore, Larkin and Thompson [70] and Smith et al [61] recommended that a meticulous, detailed, organized, plausible, and transparent account of the analytical process must be kept. This study will do so.

With respect to the overarching aim of this pragmatic study, which centers on equitable MHC, Article 1 of the 1992 United Nations’ Minorities Declaration expects the state to protect a minority’s existence [71]. In the United Kingdom, the Equality Act [72] places a statutory duty on public sectors and wider society to promote racial equality, including in MHC. The process of improving this research population’s MHC may positively impact them, their friends and associates, and their involvement in street and knife crimes in the future; it might combat stigma and reduce the negative connotations and harmful superstitions of mental health–related issues that characterize these communities; and it might contribute to a more liberal understanding that could elicit access to early intervention or the seeking of professional help. The findings of this study are expected to be published in 2025.

## Abbreviations

CAMHS: Child and Adolescent Mental Health Service
CMD: common mental disorders
IBPA: intersectionality-based policy analysis
IPA: interpretative phenomenological analysis
MHC: mental health care
NAGYP: Nigerian and Ghanaian young people
NHS: National Health Service
PPG-IAPT: Positive Practice Guide of Improving Access to Psychological Therapy
TA: thematic analysis
UCL: University College London

## Data Availability

The data sets collected and analyzed for the duration of this study will be made available from the corresponding author upon reasonable request.
